# Global prevalence of short bowel syndrome–associated intestinal failure in adults and children: A targeted literature review and analysis

**DOI:** 10.1002/ncp.11314

**Published:** 2025-05-23

**Authors:** Bilal Khokhar, Vivek Pathania, Pradeep Nazarey, Narendra Parihar

**Affiliations:** ^1^ Takeda Development Center Americas, Inc. Cambridge Massachusetts USA; ^2^ Clarivate Noida India; ^3^ Takeda Pharmaceuticals U.S.A., Inc. Cambridge Massachusetts USA; ^4^ Clarivate Philadelphia Pennsylvania USA

**Keywords:** epidemiology, home parenteral nutrition, prevalence, short bowel syndrome–associated intestinal failure

## Abstract

Short bowel syndrome–associated intestinal failure (SBS‐IF) is a rare disease caused by loss of function of the intestinal surface area and the subsequent inability to maintain nutrient and fluid balance on a normal diet, which results in the need for parenteral nutrition (PN) and/or intravenous fluids. There is a scarcity of literature regarding the prevalence of SBS‐IF and challenges in estimating prevalence. A targeted literature review was conducted to generate prevalence estimates for SBS‐IF, primarily defined by the requirement for home PN (HPN), in adults and children across multiple geographies. Country‐specific estimates of HPN prevalence and the proportion of HPN cases associated with SBS were obtained from the literature and used to inform SBS‐IF prevalence estimates. Adults were defined as those aged ≥18 years and children as those aged 0–17 years, except in Japan, where adults were those aged ≥15 years and children were aged 0–14 years. In total, 15 studies were included and were used to estimate and extrapolate prevalence across 61 countries for the years 2020–2030. The estimated prevalences of diagnosed SBS‐IF in the general population in 2024 ranged from 0.12 to 2.74 per 100,000 in adults and 0.09 to 1.67 per 100,000 in children. Prevalence estimates were generally lower in countries with a lower average income. This study provides up‐to‐date insights into the overall global and country‐by‐country prevalence of SBS‐IF and in defined cohorts of adults and children, addressing important gaps in the current literature.

## INTRODUCTION

Short bowel syndrome (SBS) is characterized in adults by the European Society for Clinical Nutrition and Metabolism (ESPEN) and the World Health Organization (WHO) as the clinical condition associated with the remaining small bowel in continuity of <200 cm.^1^, ^2^ In children, SBS has been defined by the *International Classification of Diseases, Eleventh Edition* (*ICD‐11*) (DA96.04) as <25% of the normal length of small intestine for the patient's age.^2^ The American Society for Parenteral and Enteral Nutrition (ASPEN) defines pediatric intestinal failure (IF) as “the reduction of functional intestinal mass below that which can sustain life, resulting in dependence on supplemental parenteral support for a minimum of 60 days within a 74 consecutive day interval.”^3^ In line with this definition, SBS‐associated IF (SBS‐IF) generally is defined by the loss of function of the intestinal surface area and the subsequent inability to maintain nutrient and fluid balance on an oral and/or enteral diet,^4^ resulting in the need for parenteral nutrition (PN) and/or intravenous (IV) fluid.^5^, ^6^ Definitions of SBS, however, differ between countries and societies.^7^ SBS‐IF is a rare disease that has a substantial impact on the quality of life of patients.^8^ Rare diseases pose unique challenges to patients, often relating to the availability of appropriate support and access to specialist medical care.^9^

Limited data exist on the epidemiology of SBS and SBS‐IF owing to the rarity of these diseases and inconsistency in their definitions. Furthermore, estimates for their incidence and prevalence are highly varied and outdated and can lack robust methodology.^5^, ^10^, ^11^, ^12^, ^13^

Most studies on the incidence and prevalence of SBS and SBS‐IF use home PN (HPN) as a proxy for identifying SBS‐IF.^11^ Data from 1993 indicated that the incidence of HPN among adults across eight countries in Europe ranged from 0.2 to 4.6 patients per million inhabitants per year, with a prevalence of 0.3 to 12.2 per million on January 1, 1994. The same study reported that SBS was the most common indication for HPN, making up approximately one‐third of reported cases.^10^, ^14^ A European survey conducted in 1997 estimated the prevalence of HPN to be approximately 4 per million adults in 1998; patients with SBS represented the largest group of those who required HPN (35% of identified cases).^10^, ^15^ Estimates for children were obtained in a separate study, with incidences of 0.2 to 4.9 per million per year and prevalences of 0.3 to 8.9 per million in 1998 reported across six European countries: Belgium, Denmark, France, Poland, Spain, and the UK.^16^ More recently, a European study evaluated the use of HPN among patients with IF over 40 years, with an increase in the prevalence observed between 1970 and 2010. In total, 307 of the 508 patients with IF identified between 1970 and 2010 were defined as having SBS.^17^ In 1992 in the US, there were approximately 40,000 patients who required HPN.^18^, ^19^ Because there were no specific *ICD‐10* codes for SBS and IF available in the US until they were expanded in 2023 to include codes K90.82 for SBS and K90.83 for IF,^20^, ^21^, ^22^ a previous US study used a proxy to identify SBS. The study used the *ICD‐10* code for postsurgical malabsorption, not elsewhere classified (K91.2), as well as either an additional diagnosis of K91.2 during the study period or at least two parenteral support claims during the study as a proxy for SBS. The study estimated there to be approximately 12,000 patients with durable malabsorption in 2019, defined by a diagnosis of postsurgical malabsorption 12–15 months before or following the index claim or with at least two claims for parenteral support during the study period.^23^

Furthermore, estimates for the prevalence of ultrashort bowel syndrome, defined as small bowel length of <10 cm or <10% of the expected length for the patient's age, are limited owing to the heterogeneity of the patient group and lack of national and international data.^24^

Here, we performed a targeted literature review to inform the generation of prevalence estimates for diagnosed SBS‐IF in adults and children across multiple geographies.

## METHODS

A targeted literature review was performed to obtain country‐specific estimates of HPN prevalence and the proportion of HPN cases associated with SBS to generate estimates of the prevalence of SBS‐IF.

### Eligibility criteria

Studies were eligible for inclusion if they reported the country‐specific number of HPN cases or HPN prevalence in the general population and/or reported the frequency of SBS as the indication for HPN. Adults were defined as being aged ≥18 years old and children were defined as being aged 0–17 years, except in Japan, where adults were aged ≥15 years and children were aged 0–14 years. SBS‐IF was defined by the requirement for HPN, assumed to represent moderate‐to‐severe SBS‐IF. Therefore, the search string identified cases representing moderate‐to‐severe SBS‐IF. Clinical trials, animal studies, and in vitro studies were excluded.

### Search strategy

A targeted literature review from January 1, 2012, to February 24, 2022, was performed using PubMed; prevalence estimate generation was based on literature obtained through this review. The search strategy included search terms for the population of interest (patients with SBS) with terms related to prevalence, HPN, and study design. Studies were not restricted by language or geography. The PubMed search string is included in File S1.

Studies were screened by one reviewer (V.P.) based on the eligibility criteria; approximately 10% of all studies shortlisted were randomly reviewed by a second reviewer (N.P.) as part of a quality check process. In addition, 25% of the extracted study results were independently appraised by a second reviewer (N.P.). Any disputes were resolved by a third‐party reviewer. Studies were then appraised for reliability and representativeness of the populations of interest and were included or excluded as appropriate. The Joanna Briggs Institute criteria were used to evaluate the quality of included studies (data not shown).^25^ Given the paucity of evidence on SBS and HPN, no threshold was set for the quality evaluation.

Manual searches were also performed, which involved searches of reference lists from full‐text publications identified during screening and a web‐based search for any publicly available information relevant to the epidemiology of HPN or SBS, such as congress abstracts, documents published by government agencies and nonprofits, and news articles.

### Analyses

To calculate the number of cases of SBS‐IF in each country, the age‐specific diagnosed prevalence estimates from identified studies were multiplied by the United Nations' age‐specific population estimates for 2019.^26^ Estimates were generated and projected for the years 2020 to 2030. If no data were available for country‐specific estimates of HPN prevalence and the proportion of HPN cases associated with SBS, extrapolations were performed based on geographic proximity and/or similarity of economic background (ie, based on levels of economic development as a proxy for availability of and access to facilities for HPN) where feasible. Otherwise, the mean of the evidence from all countries with reported country‐specific prevalence or the proportion of HPN cases associated with SBS was used for extrapolation. It was assumed that country‐specific prevalence data from studies conducted in HPN centers were representative of the prevalence in the general population.

The gross national income per capita reported for each country by the World Bank was used as a proxy for the levels of economic development for countries to inform groupings by income when extrapolations were required. Lower‐income countries were defined as those with a gross national income per capita of <$13,205 in 2021.^27^ For the purposes of extrapolations, lower‐income countries included Argentina, Brazil, Bulgaria, China, Colombia, Costa Rica, Dominican Republic, Ecuador, Guatemala, Indonesia, Malaysia, Mexico, Paraguay, Peru, Philippines, Russia, Thailand, Turkey, Venezuela, and Vietnam. Evidence from high‐income European countries was used to extrapolate to other European countries if reliable country‐specific evidence was not available. For lower‐income countries, Switzerland was used for extrapolations because it had the lowest 12‐month HPN prevalence reported among high‐income countries. This was done based on the assumption that the prevalence of HPN in a given country is influenced by the quality of the healthcare system (ie, availability of and access to facilities for HPN), which is, in turn, a function of the level of economic development of a country. For Israel, a mean of the prevalence reported in all studies, except the Japanese study, was used. For the remaining countries that did not report HPN use, a mean of the prevalence reported in all studies included in the analysis, except the Japanese study, was used. The Japanese study was not included when using the mean prevalence of other studies owing to the geographic and ethnic differences between Japan and the other included countries. More information regarding extrapolation is provided in Table S1.

For descriptive statistics by region, countries were grouped as follows: Asian countries were China, Hong Kong, Indonesia, Israel, Japan, Malaysia, Philippines, Singapore, South Korea, Taiwan, Thailand, Turkey, and Vietnam; Australasian countries were Australia and New Zealand; European countries were Austria, Belgium, Bulgaria, Croatia, Cyprus, Czech Republic, Denmark, Estonia, Finland, France, Germany, Greece, Hungary, Ireland, Italy, Latvia, Lithuania, Luxembourg, Malta, the Netherlands, Poland, Portugal, Romania, Russia (considered to be a European country for the purposes of this study), Slovakia, Slovenia, Spain, Sweden, Switzerland, and the UK; North American countries were Canada and the US; Central and South American countries were Argentina, Brazil, Chile, Colombia, Costa Rica, Dominican Republic, Ecuador, Guatemala, Mexico, Panama, Paraguay, Peru, Uruguay, and Venezuela. Owing to the review‐based nature of this study, institutional review board approval was not required.

## RESULTS

### Data sources

**Figure 1:**
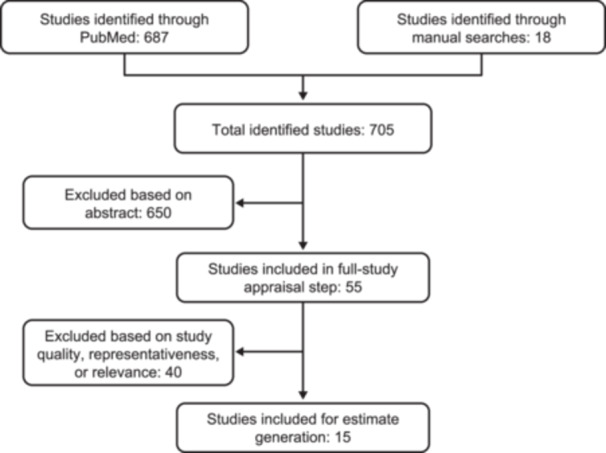
Studies identified and screened for inclusion.

Overall, 705 potentially relevant studies were identified: 687 through PubMed and 18 through manual searches (Figure 1). Studies were eligible for inclusion if they reported the country‐specific number of HPN cases or HPN prevalence in the general population and/or reported the frequency of SBS as the indication for HPN. After applying the eligibility criteria, 15 studies were included: 5 in a mixed population of adults and children, 3 in adults, 3 in children, and 4 in patients whose age was not clearly reported (Table 1). In general, included studies were analyses of health insurance claims, database studies (such as those based on Medicare data), or studies based on surveys.

**Table 1:** Details of studies reporting data on prevalence of SBS or HPN.

Citation	Data source	Data source details	Country	Period	Age/age category (years)	Denominator population (*N*)	Measure (*n*)	Disease definition of metric	Reported prevalence (*n*/*N*) or proportion (%) of SBS or HPN
Population: mixed ages or not reported									
Noelting et al.^28^	Canadian HPN Registry	Retrospective analysis of data collected from patients requiring HPN entered prospectively into the registry	Canada	2003–2017	NR	Patients requiring HPN (734)	SBS (327)	NR	44%
von Websky et al.^29^	German care centers	Randomized selection of German care centers, stratified according to the number of beds	Germany	2012	NR	General (NR)	HPN (2808)	SBS included all patients who are permanently dependent on (home) PN (also IV supplemental nutrition or hydration)	3.4/100,000
Rice et al.^30^	Literature search	Collation of relevant data obtained from a comprehensive search of the literature, expert reports, practice standards, and guidelines for good clinical practice	Ireland	2010	Adults and children	General (NR)	PN (45)	NR	1.01/100,000 SBS proportion: 28%
Takagi et al.^31^	HPN Registry Promotion Committee	The institution‐based committee was organized within the SHPN in 1989 and the first survey of the national status of the SHPN registry was carried out in 1991	Japan	1991	NR	Patients requiring HPN (231)	SBS (79)	NR	34%
Takagi^32^	Annual survey conducted from in 2000 by a commission	For the purpose of understanding the conditions of patients requiring HPN and promoting their dissemination, a commission was established in 1989 and conducts an annual survey	Japan	2000	NR	General (NR)	HPN (644)	NR	NR
Wanden‐Berghe et al.^33^	NADYA‐SENPE group registry	Descriptive analysis carried out	Spain	2019	Children (<14)	General (NR)	HPN (31)	NR	0.60/100,000 51.6% children 37.3% adults
					Adults (≥14)		HPN (252)		
Mundi et al.^34^	Medicare data were obtained from the Centers for Medicare and Medicaid Services' Medicare Referring Provider DMEPOS Data CY2013	Database with 100% of Medicare enrollment and fee‐for‐service claims data	USA	2013	Adults and children	Medicare population (NR; original study reported prevalence adjusted to the US general population using ratios of Medicare to non‐Medicare beneficiaries serviced by medical equipment providers)	In‐study population: HPN (6778); nationally projected HPN: pediatric (4129), adults (20,883)	For PN, HCPCS codes were used	7.9/100,000
Winkler et al.^35^	Sustain Registry	Descriptive analysis of data from the first cohort of patients requiring HPN at time of enrollment	USA	2011–2014	Adults (≥18)	Patients requiring HPN (adults, 1064; children, 187)	SBS	NR	24%
					Children (<18)				58%
Smith et al.^36^	BANS report	Set up as a committee of BAPEN in 1996 to collect and analyze data pertaining to enteral nutrition and PN support in adults and children in hospital and the community	UK	2010	Adults and children (children: ≤16)	General (NR)	HPN: 523 adults, 16 children	NR	–
Population: adults									
Folwarski et al.^37^	Anonymized data from the NHF	Retrospective analysis of adult patients undergoing HPN treated in Poland between January 1, 2010, and December 31, 2020	Poland	2010–2020	Adults (≥18)	General (NR)	HPN (2038)	Indications for HPN and comorbid diseases described with the *ICD‐10* coding were collected	5.326/100,000 in 2020
Reber et al.^38^	Prospective multicenter observational study	Questionnaires distributed to patients and their treating physicians in charge of PN	Switzerland	2017–2019	Adults (≥18)	General(6,835,622)	HPN (70)	NR	0.5/100,000 adult inhabitants in 2019 SBS proportion: 30%
Smith and Naghibi^39^	BANS report	Set up as a committee of BAPEN in 1996 to collect and analyze data pertaining to enteral nutrition and PN support in adults and children in hospital and the community	UK	2015	Adults	Patients requiring HPN (1144)	SBS (531)	NR	46%
Population: children									
Wyszomirska et al.^40^	Pediatric patients who underwent home enteral nutrition and PN procedures in Poland	Retrospective analysis	Poland	2010–2018	Children (0–18)	General (NR)	HPN (264)	NR	3.81/100,000 in 2018
Wiskin et al.^41^	Pediatric e‐BANS	Established in 2015 to provide an accurate contemporary database of nutrition support, including children requiring HPN in the UK	UK	2015–2019	Children (<18)	General (NR)	HPN (525)	NR	3/100,000 children in 2019 SBS proportion: 48%
Lezo et al.^42^	SIGENP and SIP member survey; data collected from 22 centers	Hospitals and institutes involved in the survey are authorized to perform research and clinical studies by the Ministry of Health	Italy	2016	Children (0–19)	General (NR)	HPN	NR	1.58/100,000

Abbreviations: BANS, British Artificial Nutrition Society; BAPEN, British Association for Parenteral and Enteral Nutrition; CY, calendar year; DMEPOS, Durable Medical Equipment, Prosthetics, Orthotics and Supplies; e‐BANS, electronic BANS; HCPCS, Healthcare Common Procedure Coding System; HPN, home parenteral nutrition; *ICD‐10*, *International Classification of Diseases, Tenth Revision*; IV, intravenous; NHF, National Health Fund; NR, not reported; PN, parenteral nutrition; SBS, short bowel syndrome; SHPN, Society for Home Parenteral Nutrition; SIGENP, Società Italiana di Gastroenterologia, Epatologia e Nutrizione Pediatrica; SIP, Società Italiana di Pediatria.

HPN prevalence was reported by 11 studies (Table S2). Most sources covered Europe (9 studies); 1 study each was identified for Japan and North America. Of the studies reporting HPN prevalence, 5 were based on surveys from HPN centers, 1 from Medicare data, and 5 from national registries/surveys (Table 1).

### Estimates and projections of prevalence of SBS‐IF for 2020 to 2030

**Figure 2:**
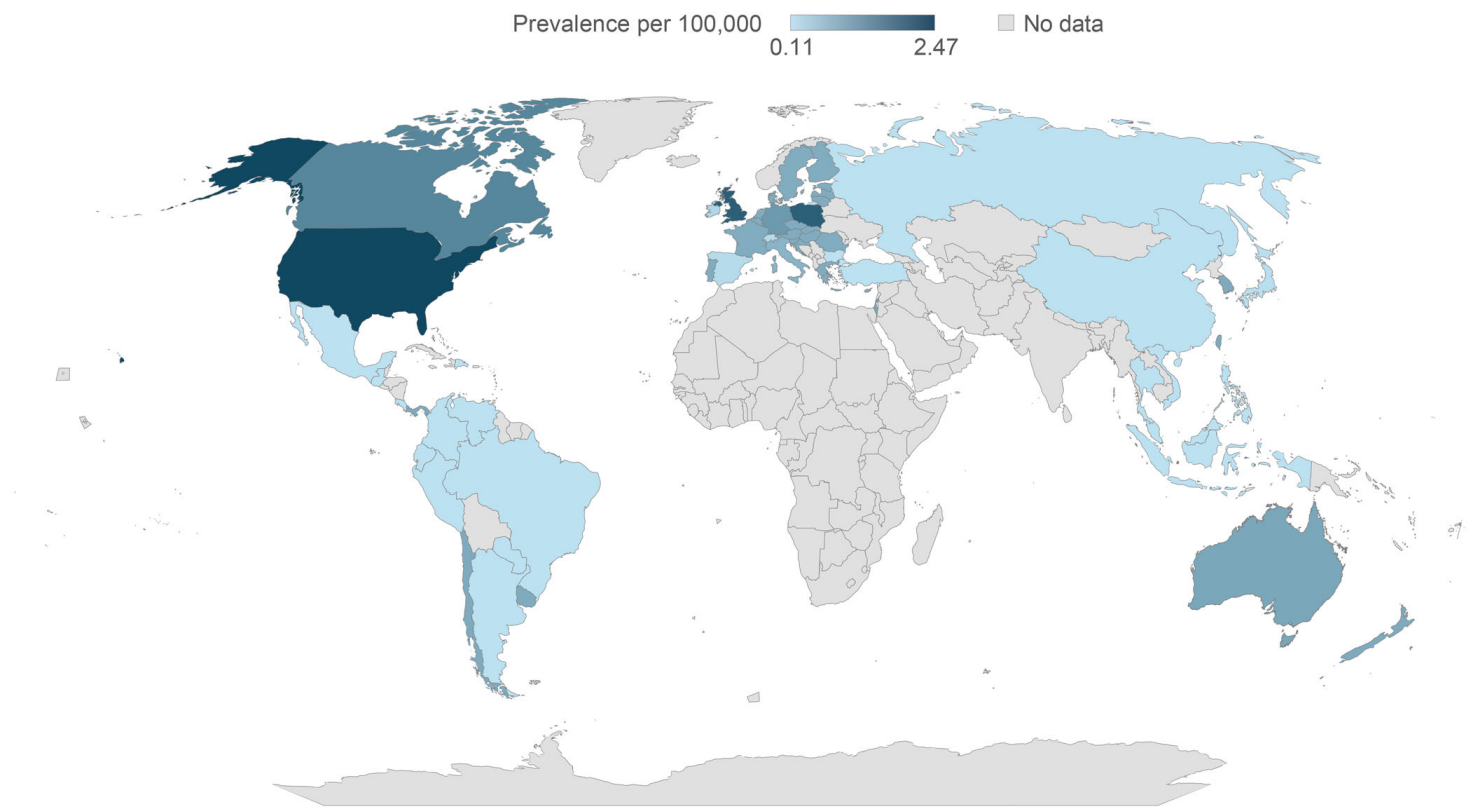
Estimated prevalence of short bowel syndrome–associated intestinal failure in adults and children per 100,000 in 2024.

**Figure 3:**
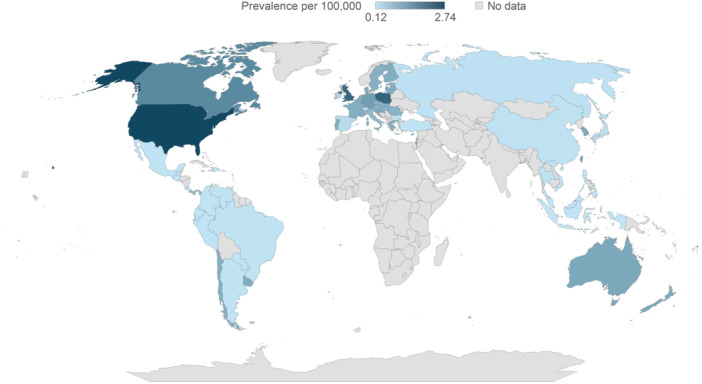
Estimated prevalence of short bowel syndrome–associated intestinal failure in adults per 100,000 in 2024.

**Figure 4:**
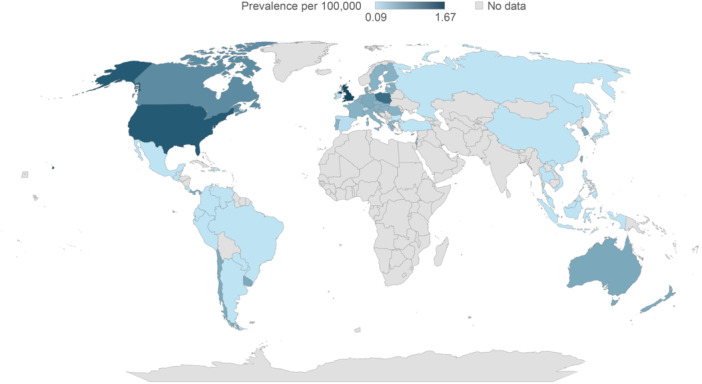
Estimated prevalence of short bowel syndrome–associated intestinal failure in children per 100,000 in 2024.

Extrapolation, based on the mean of countries of a similar income and region, was required for several countries (Table S1 and Table S3). Prevalence of SBS‐IF in the general population was estimated and projected for adults and children, both as individual and combined cohorts, across 61 countries globally for the years 2020 to 2030 (Figures 2, 3, 4, Table 2, and Tables S4 and S5); within the Results, we focus on data for 2024. No reliable identified literature presented risk factors for HPN that may be expected to change over the course of the study forecast period in a quantifiable manner. Therefore, in the absence of any supporting and quantifiable evidence of a changing risk of HPN over the forecast period, the prevalence was held constant within each age category, and changes in the number of prevalent cases over the forecast period were because of changes in the size and composition of the overall population.

**Table 2:** Estimated prevalence of short bowel syndrome–associated intestinal failure in adults and children per 100,000 from 2020 to 2030.

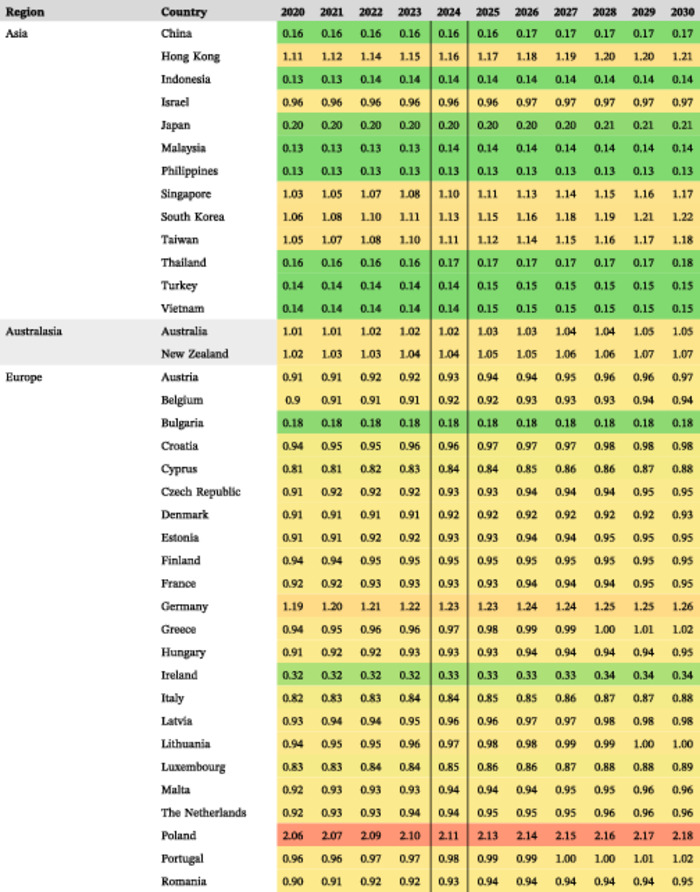 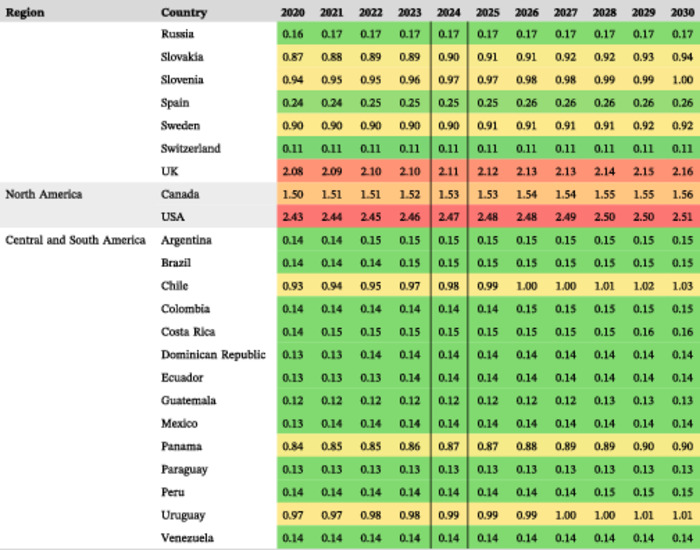

*Note*: Prevalence values are shown as lowest (green), middle (yellow; 50th percentile), and highest (red).

#### Estimated prevalence of SBS‐IF overall in 2024

The estimated mean prevalences of SBS‐IF overall in 2024 were lowest across countries in Central and South America (0.31 per 100,000; 14 countries) and highest across countries in North America (2.00 per 100,000; two countries; Figure 2). The highest estimated prevalence of SBS‐IF was in the US (2.47 per 100,000) followed by Poland and the UK (each 2.11 per 100,000). The lowest prevalence estimates were generally in countries with the lowest income. The estimated prevalences increased numerically for most countries between 2020 and 2030 (Table 2).

#### Estimated prevalence of SBS‐IF in adults in 2024

Across countries, the estimated prevalences of SBS‐IF in adults in 2024 ranged from 0.12 to 2.74 per 100,000 (Figure 3). Overall, the prevalences of SBS‐IF in adults were lowest across countries in Central and South America (mean prevalence 0.33 per 100,000; 14 countries) and highest across countries in North America (2.19 per 100,000; two countries). By region, the range of prevalences were: 0.13 (Philippines) to 1.24 (Hong Kong) in Asia; 1.12 (Australia) to 1.14 (New Zealand) in Australasia; 0.12 (Switzerland) to 2.29 (Poland) in Europe; 1.65 (Canada) to 2.74 (US) in North America; and 0.12 (Guatemala) to 1.07 (Uruguay) in Central and South America. The estimated prevalences increased numerically for most countries between 2020 and 2030 (Table S4).

#### Estimated prevalence of SBS‐IF in children in 2024

The estimated prevalence of SBS‐IF in children in 2024 ranged from 0.09 to 1.67 per 100,000 across all countries included in the study (Figure 4). Similar to adults, generally the mean prevalences of SBS‐IF in children were lowest across countries in Central and South America (0.26 per 100,000; 14 countries) and highest across countries in North America (1.24 per 100,000; two countries). The range of prevalences were 0.11 (Japan) to 0.74 (Israel) in Asia; 0.71 for both Australia and New Zealand in Australasia; 0.09 (Switzerland) to 1.67 (UK) in Europe; 0.99 (Canada) to 1.49 (US) in North America; and 0.14 (Argentina, Brazil, Colombia, Costa Rica, Dominican Republic, Ecuador, Guatemala, Mexico, Paraguay, Peru, and Venezuela) to 0.71 (Chile, Panama, and Uruguay) in Central and South America. The estimated prevalences did not change between 2020 and 2030 (Table S5).

## DISCUSSION

The incidence and prevalence of SBS‐IF is not well understood owing to the rarity of the disease, varied definitions of SBS‐IF, underreporting, and a lack of reliable datasets (eg, studies may use surveys of clinicians who have few or no patients with SBS‐IF).^5^, ^10^, ^11^, ^12^, ^13^ In addition, no *ICD‐9* or *ICD‐10* codes were available for SBS or IF in the US at the time of these studies^43^, ^44^, ^45^ or the present study.^20^, ^21^, ^22^ Awareness and understanding of SBS‐IF among healthcare professionals have been identified as areas that may need improvements,^46^ and as such, robust and up‐to‐date prevalence estimates may help to improve these aspects and thus enhance patient care. To our knowledge, this study is the first of its kind in SBS‐IF and reports comprehensive, up‐to‐date global estimates for the prevalence of SBS‐IF in adults and children based on data obtained through a targeted literature review.

The estimated global prevalences we have reported here, ranging from 0.12 to 2.74 per 100,000 in adults and 0.09 to 1.67 per 100,000 in children in 2024, are generally in line with previous reports that were also based on HPN or PN/IV use.^5^, ^10^, ^11^, ^12^, ^13^ A review from 2020 presented prevalence estimates of adults with SBS‐IF from previous studies, many of which were conducted >15 years ago. These studies reported prevalences of approximately 0.04 to 4 cases per 100,000 in Europe in 1993 and 3 cases per 100,000 in the US in 1992.^12^ For most countries, prevalence estimates were higher in adults than children. In addition, we found that the upper range of estimated prevalence was higher for adults than children although the overall trend in country‐specific estimates was similar. These observations may be explained by the nature of the analysis performed in the current study (ie, the prevalence estimates take into account the overall country‐specific prevalence of HPN, the extrapolated age‐distribution of HPN, and the country‐specific demographic profile). These observations should, however, be made in consideration of the fact that extrapolation was required to obtain estimates of prevalence for many countries and for certain age groups within countries.

Our results demonstrate that the prevalence of SBS‐IF varies globally, which was also found in previous observations,^12^ and is likely to be higher in developed countries. The reason for the lower prevalence rates estimated in lower‐income countries may relate to a lack of infrastructure for HPN^47^ as well as a lack of major intestinal rehabilitation centers, affecting underreporting in such regions.^48^

Limited evidence from the literature was identified that suggested historical trends in prevalence of diagnosed SBS; one study was identified that reported an increase in the annual prevalence of patients with IF who were receiving HPN between 1970 and 2010.^17^ As such, within the present study, the estimated prevalence was not projected to increase to a great extent between 2020 and 2030, and any estimated increase was based only on a general increase in the population size. The introduction of *ICD‐10* codes for SBS and IF^20^, ^21^, ^22^ may help to create consistency in diagnosis reporting and therefore provide more robust estimates in the US;^49^ however, there are currently not enough data available to use these codes.

The present study has several strengths. Primarily, this is a comprehensive, multinational study that provides prevalence estimates from a wide range of countries across the globe for adults and children combined and as separate cohorts. We used innovative methods to generate estimates that are informative and may be closer to actual values than previous studies have generated. Furthermore, we generated estimates for countries and regions where there may be little infrastructure for HPN and data on SBS‐IF and, thus, limited published data on prevalence. Additionally, this study was able to generate projections of prevalence up to 2030 and address a key evidence gap for prevalence estimates in this rare disease.

The strengths of the study notwithstanding, there are some limitations that must be considered when interpreting the results. The literature review was targeted rather than systematic. Although the methodology is fully described and, as such, is not a narrative review, it is possible that some relevant literature may not have been identified during the review process. The proxies used may result in underestimation or overestimation of prevalence; however, to counter this limitation, proxies were selected to represent countries with a similar economic background. Extrapolation was required to obtain values for several countries, particularly those that may have limited healthcare infrastructure for HPN, which may also result in underestimation or overestimation of prevalence. The potential lack of funding or reimbursement for HPN in such countries may impact on access to HPN and therefore on prevalence estimates.^50^, ^51^ However, no studies were identified and used to base estimates and extrapolations on from lower‐income countries; thus, we did not anticipate a great impact of this on our results. Furthermore, HPN use is likely to represent the outpatient setting, with patients generally receiving HPN at home and attending HPN centers as required, the availability of which may differ by region,^4^, ^52^ and, as such, the prevalence of patients who are managed only in the inpatient setting has not been captured. In addition, no adjustments were made to address any differences in the duration of HPN reported by studies. Lastly, in the absence of robust, up‐to‐date estimates of the incidence or prevalence of SBS‐IF,^12^ estimates were made based on proxies such as HPN use. This method of estimation introduces a further potential area of uncertainty owing to the heterogeneity in methods of reporting (eg, point prevalence vs period prevalence^11^) and may lead to difficulty in making comparisons between studies. It should be noted, however, that the current study made adjustments where relevant to standardize the HPN prevalence estimates reported by the included studies to a 12‐month prevalence measure.^52^ However, HPN use has previously been used as a proxy for SBS‐IF prevalence.^5^, ^10^, ^11^, ^12^, ^13^

## CONCLUSIONS

This novel study provides comprehensive up‐to‐date insights into the overall global and country‐by‐country prevalence of a rare disease, SBS‐IF, as well as in defined cohorts of adults and children, and it gives insights into the potential future burden of the disease. The results presented here therefore address important gaps in the current literature. To our knowledge, no study has published prevalence estimates for SBS‐IF for adults and children separately. Furthermore, no previous study has provided SBS‐IF prevalence estimates and projected prevalence estimates for 61 countries. Our results are broadly in line with previously published (but outdated) estimates, and they are based on generally robust methodology. Overall, estimated annual prevalences of SBS‐IF were highest in Poland, the UK, and the US although they were within a relatively small range globally. Furthermore, we do not predict the prevalence of SBS‐IF to increase to a great extent before 2030, thus suggesting that SBS‐IF will remain a rare disease and will likely retain some of the challenges associated with the diagnosis and treatment of a rare disease. Future work should focus on generating evidence that will enable prevalence estimates of SBS‐IF to be obtained for developing countries, where infrastructure for HPN may be poor.

## AUTHOR CONTRIBUTIONS

Bilal Khokhar, Vivek Pathania, and Narendra Parihar equally contributed to the conception and design of the research. Vivek Pathania and Narendra Parihar contributed to the acquisition and analysis of the data. All authors contributed to the interpretation of the data, critically revised the manuscript, and agree to be fully accountable for ensuring the integrity and accuracy of the work, and read and approved the final manuscript.

## CONFLICT OF INTEREST STATEMENT

Bilal Khokhar is an employee of Takeda and receives stock and/or stock options. Pradeep Nazarey was an employee of Takeda at the time of the study and received stock and/or stock options. Vivek Pathania and Narendra Parihar are employees of Clarivate, and their contributions to this study were in part funded by Takeda.

## Supporting information

SBS Epidemiology SUPPLEMENTARY REVISED 6Mar25.
