# Supplementary material for: Global prevalence of short bowel syndrome–associated intestinal failure in adults and children: A targeted literature review and analysis
Source: Nutr Clin Pract. 2025 May 23;40(5):1093–106. doi: 10.1002/ncp.11314 (PMC12450332; doi:10.1002/ncp.11314)
Supplement: Supplementary file 1 — SBS Epidemiology SUPPLEMENTARY REVISED 6Mar25. [file NCP-40-1093-s001.pdf]

Supplementary Materials

PubMed search string

(short bowel syndrome[tiab] OR small bowel resection[tiab] OR short bowel resection[tiab]) AND (inciden*[tiab] OR prevalen*[tiab] OR risk*[tiab] OR frequen*[tiab] OR trend*[tiab] OR treat*[tiab] OR survival[tiab] OR injur*[tiab] OR parenteral[tiab] OR intravenous [tiab] OR sever*[tiab] OR mild[tiab] OR moderate[tiab] OR anatom*[tiab] OR jejuno*[tiab] OR pathophysiolog*[tiab] OR early[tiab] OR adapt*[tiab] OR maintenance[tiab] OR clinical[tiab]) AND ("Cross-Sectional"[tiab] OR cohort[tiab] OR "Follow-Up"[tiab] OR prospective[tiab] OR retrospective[tiab] OR longitudinal[tiab] OR observational[tiab] OR "population based"[tiab] OR "population-based"[tiab] OR nationwide[tiab] OR national[tiab] OR hospital-based[tiab] OR clinic[tiab] OR regist*[tiab] OR Epidemiol*[tiab] OR database*) AND (2012:2022[PDAT]) Filters: Abstract.

Table S1 Extrapolations required for prevalence estimates.

	Countries used for extrapolation	
**Country of interest**	**HPN prevalence**	**SBS among HPN cases**
**France**	Mean of Ireland, Italy, Germany, Poland, Spain, Switzerland, and UK	Mean of Ireland, Spain, Switzerland[a], and UK
**Germany and Italy**	NA[b]	Mean of Ireland, Spain, Switzerland[a], and UK
**Ireland**	NA[b]	NA[b]
**Poland**	NA[b]	Mean of Ireland, Spain, Switzerland[a], and UK
**Switzerland**	NA[b]	NA[a,b]
**Rest of the European countries[c]**	Mean of Germany, Italy, Spain, Switzerland, Poland, Ireland, and UK	Mean of Ireland, Spain, Switzerland[a], and UK
**Canada**	Mean of Germany, Ireland, Italy, Japan, Poland, Spain, Switzerland, UK, and USA	NA[b]
**Israel**	Mean of Germany, Ireland, Italy, Poland, Spain, Switzerland, UK, and USA	Mean of Ireland, Spain, Switzerland[a], UK, and USA
**Japan**	NA[b]	NA[b]
**USA**	NA[b]	NA[b]
**Lower-income countries[d]**	Switzerland	Mean of Canada, Ireland, Japan, Spain, Switzerland[a], UK, and USA
**Remaining countries[e]**	Mean of Germany, Ireland, Italy, Japan, Poland, Spain, Switzerland, UK, and USA	Mean of Canada, Ireland, Japan, Spain, Switzerland[a], UK, and USA

Abbreviations: HPN, home parenteral nutrition; NA, not applicable; SBS, short bowel syndrome.

[a]SBS proportion among HPN use was reported only for adults in Switzerland[1] and was adjusted to reflect the proportion for all ages using data from Spain.[2]

[b]Country-specific data available.

[c]Rest of the European countries were Austria, Belgium, Cyprus, Croatia, Czech republic, Denmark, Estonia, Finland, Greece, Hungary, Latvia, Lithuania, Luxembourg, Malta, Netherlands, Portugal, Romania, Slovakia, Slovenia, Spain, Sweden, and United Kingdom.

[d]Lower-income countries were Argentina, Brazil, Bulgaria, China, Colombia, Costa Rica, Dominican Republic, Ecuador, Guatemala, Indonesia, Malaysia, Mexico, Paraguay, Peru, Philippines, Russia, Thailand, Turkey, Venezuela, and Vietnam.

[e]Remaining countries were Australia, Chile, Hong Kong, New Zealand, Panama, Singapore, South Korea, Taiwan, and Uruguay.

Table S2 Availability of country-specific data on HPN prevalence with or without SBS proportion among included studies.

Abbreviations: HPN, home parenteral nutrition; SBS, short bowel syndrome.

Country	Citation	HPN prevalence (from literature)	SBS proportion among HPN cases (from literature)
Canada	Noelting, 2021[3]	x	✓
Germany	von Websky, 2014[4]	✓	x
Ireland	Rice, 2013[5]	✓	✓
Italy	Lezo, 2018[6]	✓	x
Japan	Takagi, 2003[7]	✓	x
	Takagi, 1995[8]	x	✓
Poland	Folwarski, 2021[9]	✓	x
	Wyszomirska, 2021[10]	✓	x
Spain	Wanden-Berghe, 2021[2]	✓	✓
Switzerland	Reber, 2021[1]	✓	✓
UK	Smith, 2011[11]	✓	x
	Smith, 2016[12]	x	✓
	Wiskin, 2021[13]	✓	✓
USA	Mundi, 2017[14]	✓	x
	Winkler, 2016[15]	x	✓

Table S3 Sources and methods used to estimate SBS-IF prevalence in the general population.

Country	Citation	HPN prevalence per 100,000	Adjustment	Estimated age-specific HPN prevalence per 100,000[a]		Proportion of SBS-IF cases among HPN use reported in the literature applied to HPN prevalence for generating estimates
				Children	Adults	
Germany	von Websky, 2014[4]	3.4 (all ages)	Age-standardization extrapolation model to estimate age distribution, using age distribution from Poland[9,10]	2.092	3.697	Mean SBS from Ireland, Spain, Switzerland, and UK[1,2,12,13]
Ireland	Rice, 2013[5]	1.01 (all ages)	Back-calculated age-specific HPN prevalence from given HPN cases: 7 (children), 38 (adults) Age-standardization extrapolation model to estimate adult age distribution, using age distribution from Poland[9]	0.5758	1.1309	SBS proportion for adults and children from IrSPEN special report 2013: 28%[5]
Italy	Lezo, 2018[6]	1.58 (0–19 years)	Adjusted point prevalence to period prevalence from UK. Estimated pediatric prevalence using age-specific prevalence ratio (children:adults) from Spanish study[2] Age-standardization extrapolation model to estimate adult age distribution, using age distribution from Poland[9]	1.885	2.395	Mean SBS proportion for adults and children from Ireland, Spain, Switzerland, and UK[1,2,12,13]
Spain	Wanden-Berghe, 2021[2]	0.601 (all ages)	Back-calculated age-specific HPN prevalence from given HPN cases: 31 (0–13 years), 252 (≥14 years) Age-standardization extrapolation model to estimate adult age distribution, using age distribution from Poland[9]	0.490	0.623	SBS proportion: 39%[2]
USA	Mundi, 2017[14]	7.9 (all ages)	Back-calculated age-specific HPN prevalence from given HPN cases: 4129 (children), 20,883 (adults) Age-standardization extrapolation model to estimate adult age distribution, using age distribution from Poland[9]	5.215	8.719	SBS proportion: 29%[15]
UK	Wiskin, 2021[13]	3 (children point prevalence)	Adjusted children point prevalence to period prevalence from BANS report 2010. Estimated adult prevalence using age-specific prevalence ratio (children:adults) from Spanish study[2]	3.579	4.548	SBS proportion: 47%[12,13]
	Smith, 2016[12]	1.77 (adults)				

Country	Citation	HPN prevalence per 100,000	Adjustment	Estimated age-specific HPN prevalence per 100,000[a]		Proportion of SBS-IF cases among HPN use reported in the literature applied to HPN prevalence for generating estimates
				Children	Adults	
			Age-standardization extrapolation model to estimate adult age distribution, using age distribution from Poland[9]			
Switzerland	Reber, 2021[1]	0.5 (≥18 years)	Estimated children prevalence using age-specific prevalence ratio (children:adults) from Spanish study[2]<br>Age-standardization extrapolation model to estimate adult age distribution, using age distribution from Poland[9]	0.3935	0.5	SBS proportion among HPN use was reported only for adults in Switzerland and was adjusted to reflect the proportion for all ages using data from Spain: 22%[1,2]
Japan	Takagi, 2003[7]	0.505 (all ages)	Age-standardization extrapolation model to estimate age distribution, using age distribution from Polish study[9,10]	0.3293	0.5385	SBS proportion for adults and children: 34%[8]
Poland	Folwarski, 2021[9]	5.326 (adults)	NA	3.81	6.557	Averaged SBS proportion for adults and children from Ireland, Spain, Switzerland, and UK[1,2,5,12,13]
Poland	Wyszomirska, 2021[10]	3.81 (0–18 years)	NA			

Abbreviations: BANS, British Artificial Nutrition Survey; HPN, home parenteral nutrition; IrSPEN; Irish Society for Clinical Nutrition and Metabolism; NA, not applicable; SBS, short bowel syndrome; SPS-IF, short bowel syndrome-associated intestinal failure.

Table S4 Estimated prevalences of SBS-IF in adults per 100,000 from 2020 to 2030.

Region	Country	2020	2021	2022	2023	2024	2025	2026	2027	2028	2029	2030
Asia	China	0.16	0.16	0.16	0.17	0.17	0.17	0.17	0.18	0.18	0.18	0.18
	Hong Kong	1.18	1.20	1.21	1.23	1.24	1.26	1.27	1.28	1.29	1.30	1.31
	Indonesia	0.13	0.13	0.14	0.14	0.14	0.14	0.14	0.14	0.14	0.15	0.15
	Israel	1.06	1.06	1.06	1.06	1.07	1.07	1.07	1.07	1.07	1.07	1.08
	Japan	0.21	0.21	0.21	0.21	0.22	0.22	0.22	0.22	0.22	0.22	0.22
	Malaysia	0.13	0.13	0.13	0.13	0.14	0.14	0.14	0.14	0.14	0.14	0.14
	Philippines	0.12	0.12	0.13	0.13	0.13	0.13	0.13	0.13	0.13	0.13	0.13
	Singapore	1.09	1.11	1.13	1.15	1.16	1.18	1.20	1.21	1.23	1.24	1.25
	South Korea	1.13	1.15	1.16	1.18	1.20	1.22	1.24	1.25	1.27	1.29	1.30
	Taiwan	1.12	1.13	1.15	1.17	1.18	1.20	1.21	1.23	1.24	1.25	1.26
	Thailand	0.16	0.17	0.17	0.17	0.17	0.18	0.18	0.18	0.18	0.18	0.18
	Turkey	0.14	0.14	0.14	0.15	0.15	0.15	0.15	0.15	0.15	0.15	0.16
	Vietnam	0.14	0.14	0.14	0.14	0.15	0.15	0.15	0.15	0.15	0.16	0.16
Australasia	Australia	1.10	1.10	1.11	1.11	1.12	1.12	1.13	1.13	1.14	1.14	1.15
	New Zealand	1.12	1.13	1.13	1.14	1.14	1.15	1.15	1.16	1.16	1.17	1.17
Europe	Austria	0.97	0.97	0.98	0.99	1.00	1.01	1.01	1.02	1.03	1.04	1.04
	Belgium	0.97	0.98	0.98	0.99	0.99	1.00	1.00	1.01	1.01	1.01	1.02
	Bulgaria	0.19	0.19	0.19	0.19	0.19	0.19	0.19	0.19	0.19	0.19	0.19
	Croatia	1.01	1.01	1.02	1.03	1.03	1.04	1.04	1.04	1.05	1.05	1.05
	Cyprus	0.85	0.86	0.87	0.88	0.89	0.90	0.90	0.91	0.92	0.92	0.93
	Czech Republic	0.98	0.99	0.99	0.99	1.00	1.00	1.01	1.01	1.02	1.02	1.03
	Denmark	0.98	0.98	0.98	0.99	0.99	0.99	0.99	0.99	1.00	1.00	1.00
	Estonia	0.98	0.99	0.99	1.00	1.00	1.01	1.01	1.02	1.02	1.02	1.03
	Finland	1.02	1.02	1.02	1.02	1.02	1.02	1.02	1.02	1.02	1.02	1.02
	France	1.00	1.00	1.01	1.01	1.01	1.01	1.02	1.02	1.02	1.03	1.03
	Germany	1.29	1.30	1.31	1.32	1.33	1.34	1.35	1.35	1.36	1.37	1.37
	Greece	1.01	1.01	1.02	1.03	1.04	1.05	1.05	1.06	1.07	1.07	1.08
	Hungary	0.97	0.98	0.98	0.99	0.99	1.00	1.00	1.01	1.01	1.01	1.01
	Ireland	0.37	0.37	0.37	0.37	0.38	0.38	0.38	0.38	0.38	0.39	0.39
	Italy	0.86	0.86	0.87	0.87	0.88	0.89	0.89	0.90	0.90	0.91	0.92

	Latvia	1.01	1.01	1.02	1.03	1.04	1.05	1.05	1.06	1.06	1.07	1.07
	Lithuania	1.01	1.02	1.03	1.04	1.05	1.06	1.07	1.07	1.08	1.09	1.09
	Luxembourg	0.88	0.88	0.89	0.90	0.90	0.91	0.92	0.93	0.93	0.94	0.95
	Malta	0.99	0.99	0.99	1.00	1.00	1.01	1.01	1.02	1.02	1.03	1.03
	Netherlands	1.00	1.00	1.01	1.01	1.01	1.02	1.02	1.03	1.03	1.04	1.04
	Poland	2.22	2.24	2.26	2.28	2.29	2.31	2.32	2.33	2.34	2.36	2.37
	Portugal	1.02	1.03	1.03	1.04	1.04	1.05	1.06	1.06	1.07	1.08	1.09
	Romania	0.97	0.97	0.98	0.99	1.00	1.01	1.01	1.01	1.01	1.01	1.02
	Russia	0.17	0.17	0.18	0.18	0.18	0.18	0.18	0.18	0.18	0.18	0.18
	Slovakia	0.93	0.94	0.95	0.96	0.96	0.97	0.98	0.99	0.99	1.00	1.01
	Slovenia	1.01	1.02	1.03	1.03	1.04	1.05	1.06	1.06	1.06	1.07	1.07
	Spain	0.25	0.26	0.26	0.26	0.26	0.27	0.27	0.27	0.27	0.28	0.28
	Sweden	0.97	0.97	0.97	0.98	0.98	0.98	0.98	0.99	0.99	0.99	0.99
	Switzerland	0.11	0.11	0.11	0.11	0.12	0.12	0.12	0.12	0.12	0.12	0.12
	UK	2.19	2.20	2.21	2.22	2.23	2.24	2.25	2.26	2.26	2.27	2.28
North America	Canada	1.62	1.63	1.64	1.64	1.65	1.65	1.66	1.67	1.67	1.68	1.68
	USA	2.70	2.71	2.72	2.73	2.74	2.75	2.75	2.76	2.77	2.77	2.78
Central and South America	Argentina	0.15	0.15	0.15	0.15	0.15	0.15	0.15	0.15	0.15	0.15	0.15
	Brazil	0.14	0.14	0.15	0.15	0.15	0.15	0.15	0.15	0.16	0.16	0.16
	Chile	1.00	1.01	1.03	1.04	1.05	1.07	1.08	1.09	1.10	1.11	1.11
	Colombia	0.14	0.14	0.14	0.14	0.15	0.15	0.15	0.15	0.15	0.15	0.16
	Costa Rica	0.15	0.15	0.15	0.15	0.15	0.15	0.16	0.16	0.16	0.16	0.16
	Dominican Republic	0.13	0.13	0.14	0.14	0.14	0.14	0.14	0.14	0.14	0.14	0.15
	Ecuador	0.13	0.13	0.13	0.14	0.14	0.14	0.14	0.14	0.14	0.14	0.14
	Guatemala	0.11	0.11	0.11	0.11	0.12	0.12	0.12	0.12	0.12	0.12	0.12
	Mexico	0.13	0.14	0.14	0.14	0.14	0.14	0.14	0.14	0.14	0.15	0.15
	Panama	0.90	0.91	0.92	0.93	0.94	0.94	0.95	0.96	0.97	0.98	0.98
	Paraguay	0.12	0.13	0.13	0.13	0.13	0.13	0.13	0.13	0.13	0.13	0.13
	Peru	0.14	0.14	0.14	0.14	0.14	0.15	0.15	0.15	0.15	0.15	0.15
	Uruguay	1.06	1.06	1.06	1.07	1.07	1.08	1.08	1.09	1.09	1.10	1.10
	Venezuela	0.14	0.14	0.14	0.14	0.14	0.14	0.14	0.14	0.14	0.15	0.15

Abbreviation: SBS-IF, short bowel syndrome-associated intestinal failure. Prevalence values are shown as lowest, middle (50th percentile), and highest.

Table S5 Estimated prevalence of SBS-IF in children per 100,000 from 2020 to 2030.

Region	Country	2020	2021	2022	2023	2024	2025	2026	2027	2028	2029	2030
Asia	China	0.14	0.14	0.14	0.14	0.14	0.14	0.14	0.14	0.14	0.14	0.14
	Hong Kong	0.71	0.71	0.71	0.71	0.71	0.71	0.71	0.71	0.71	0.71	0.71
	Indonesia	0.14	0.14	0.14	0.14	0.14	0.14	0.14	0.14	0.14	0.14	0.14
	Israel	0.74	0.74	0.74	0.74	0.74	0.74	0.74	0.74	0.74	0.74	0.74
	Japan	0.11	0.11	0.11	0.11	0.11	0.11	0.11	0.11	0.11	0.11	0.11
	Malaysia	0.14	0.14	0.14	0.14	0.14	0.14	0.14	0.14	0.14	0.14	0.14
	Philippines	0.14	0.14	0.14	0.14	0.14	0.14	0.14	0.14	0.14	0.14	0.14
	Singapore	0.71	0.71	0.71	0.71	0.71	0.71	0.71	0.71	0.71	0.71	0.71
	South Korea	0.71	0.71	0.71	0.71	0.71	0.71	0.71	0.71	0.71	0.71	0.71
	Taiwan	0.71	0.71	0.71	0.71	0.71	0.71	0.71	0.71	0.71	0.71	0.71
	Thailand	0.14	0.14	0.14	0.14	0.14	0.14	0.14	0.14	0.14	0.14	0.14
	Turkey	0.14	0.14	0.14	0.14	0.14	0.14	0.14	0.14	0.14	0.14	0.14
	Vietnam	0.14	0.14	0.14	0.14	0.14	0.14	0.14	0.14	0.14	0.14	0.14
Australasia	Australia	0.71	0.71	0.71	0.71	0.71	0.71	0.71	0.71	0.71	0.71	0.71
	New Zealand	0.71	0.71	0.71	0.71	0.71	0.71	0.71	0.71	0.71	0.71	0.71
Europe	Austria	0.62	0.62	0.62	0.62	0.62	0.62	0.62	0.62	0.62	0.62	0.62
	Belgium	0.62	0.62	0.62	0.62	0.62	0.62	0.62	0.62	0.62	0.62	0.62
	Bulgaria	0.14	0.14	0.14	0.14	0.14	0.14	0.14	0.14	0.14	0.14	0.14
	Croatia	0.62	0.62	0.62	0.62	0.62	0.62	0.62	0.62	0.62	0.62	0.62
	Cyprus	0.62	0.62	0.62	0.62	0.62	0.62	0.62	0.62	0.62	0.62	0.62
	Czech Republic	0.62	0.62	0.62	0.62	0.62	0.62	0.62	0.62	0.62	0.62	0.62
	Denmark	0.62	0.62	0.62	0.62	0.62	0.62	0.62	0.62	0.62	0.62	0.62
	Estonia	0.62	0.62	0.62	0.62	0.62	0.62	0.62	0.62	0.62	0.62	0.62
	Finland	0.62	0.62	0.62	0.62	0.62	0.62	0.62	0.62	0.62	0.62	0.62
	France	0.62	0.62	0.62	0.62	0.62	0.62	0.62	0.62	0.62	0.62	0.62
	Germany	0.71	0.71	0.71	0.71	0.71	0.71	0.71	0.71	0.71	0.71	0.71
	Greece	0.62	0.62	0.62	0.62	0.62	0.62	0.62	0.62	0.62	0.62	0.62
	Hungary	0.62	0.62	0.62	0.62	0.62	0.62	0.62	0.62	0.62	0.62	0.62
	Ireland	0.16	0.16	0.16	0.16	0.16	0.16	0.16	0.16	0.16	0.16	0.16
	Italy	0.64	0.64	0.64	0.64	0.64	0.64	0.64	0.64	0.64	0.64	0.64

	Latvia	0.62	0.62	0.62	0.62	0.62	0.62	0.62	0.62	0.62	0.62	0.62
	Lithuania	0.62	0.62	0.62	0.62	0.62	0.62	0.62	0.62	0.62	0.62	0.62
	Luxembourg	0.62	0.62	0.62	0.62	0.62	0.62	0.62	0.62	0.62	0.62	0.62
	Malta	0.62	0.62	0.62	0.62	0.62	0.62	0.62	0.62	0.62	0.62	0.62
	Netherlands	0.62	0.62	0.62	0.62	0.62	0.62	0.62	0.62	0.62	0.62	0.62
	Poland	1.29	1.29	1.29	1.29	1.29	1.29	1.29	1.29	1.29	1.29	1.29
	Portugal	0.62	0.62	0.62	0.62	0.62	0.62	0.62	0.62	0.62	0.62	0.62
	Romania	0.62	0.62	0.62	0.62	0.62	0.62	0.62	0.62	0.62	0.62	0.62
	Russia	0.14	0.14	0.14	0.14	0.14	0.14	0.14	0.14	0.14	0.14	0.14
	Slovakia	0.62	0.62	0.62	0.62	0.62	0.62	0.62	0.62	0.62	0.62	0.62
	Slovenia	0.62	0.62	0.62	0.62	0.62	0.62	0.62	0.62	0.62	0.62	0.62
	Spain	0.19	0.19	0.19	0.19	0.19	0.19	0.19	0.19	0.19	0.19	0.19
	Sweden	0.62	0.62	0.62	0.62	0.62	0.62	0.62	0.62	0.62	0.62	0.62
	Switzerland	0.09	0.09	0.09	0.09	0.09	0.09	0.09	0.09	0.09	0.09	0.09
	UK	1.67	1.67	1.67	1.67	1.67	1.67	1.67	1.67	1.67	1.67	1.67
North America	Canada	0.99	0.99	0.99	0.99	0.99	0.99	0.99	0.99	0.99	0.99	0.99
	USA	1.49	1.49	1.49	1.49	1.49	1.49	1.49	1.49	1.49	1.49	1.49
Central and South America	Argentina	0.14	0.14	0.14	0.14	0.14	0.14	0.14	0.14	0.14	0.14	0.14
	Brazil	0.14	0.14	0.14	0.14	0.14	0.14	0.14	0.14	0.14	0.14	0.14
	Chile	0.71	0.71	0.71	0.71	0.71	0.71	0.71	0.71	0.71	0.71	0.71
	Colombia	0.14	0.14	0.14	0.14	0.14	0.14	0.14	0.14	0.14	0.14	0.14
	Costa Rica	0.14	0.14	0.14	0.14	0.14	0.14	0.14	0.14	0.14	0.14	0.14
	Dominican Republic	0.14	0.14	0.14	0.14	0.14	0.14	0.14	0.14	0.14	0.14	0.14
	Ecuador	0.14	0.14	0.14	0.14	0.14	0.14	0.14	0.14	0.14	0.14	0.14
	Guatemala	0.14	0.14	0.14	0.14	0.14	0.14	0.14	0.14	0.14	0.14	0.14
	Mexico	0.14	0.14	0.14	0.14	0.14	0.14	0.14	0.14	0.14	0.14	0.14
	Panama	0.71	0.71	0.71	0.71	0.71	0.71	0.71	0.71	0.71	0.71	0.71
	Paraguay	0.14	0.14	0.14	0.14	0.14	0.14	0.14	0.14	0.14	0.14	0.14
	Peru	0.14	0.14	0.14	0.14	0.14	0.14	0.14	0.14	0.14	0.14	0.14
	Uruguay	0.71	0.71	0.71	0.71	0.71	0.71	0.71	0.71	0.71	0.71	0.71
	Venezuela	0.14	0.14	0.14	0.14	0.14	0.14	0.14	0.14	0.14	0.14	0.14

Abbreviation: SBS-IF, short bowel syndrome-associated intestinal failure. Prevalence values are shown as lowest, middle (50th percentile), and highest.

References

1. Reber, E, K Staub *et al*. Management of Home Parenteral Nutrition: Complications and Survival. *Ann Nutr Metab.* 2021;77:46–55.
2. Wanden-Berghe, C, N Virgili Casas *et al*. [Home and Ambulatory Artificial Nutrition (NADYA) Group report: home parenteral nutrition in Spain, 2019]. *Nutr Hosp.* 2021;38:1304–1309.
3. Noelting, J, L Gramlich *et al*. Survival of Patients With Short-Bowel Syndrome on Home Parenteral Nutrition: A Prospective Cohort Study. *JPEN J Parenter Enteral Nutr.* 2021;45:1083–1088.
4. von Websky, MW, U Liermann *et al*. [Short bowel syndrome in Germany. Estimated prevalence and standard of care]. *Chirurg.* 2014;85:433–9.
5. Rice, N and J Dowsett (2013) *A Review of Home Parenteral Nutrition in Ireland: Recommendations for Action*.
6. Lezo, A, T Capriati *et al*. Paediatric Home Artificial Nutrition in Italy: Report from 2016 Survey on Behalf of Artificial Nutrition Network of Italian Society for Gastroenterology, Hepatology and Nutrition (SIGENP). *Nutrients.* 2018;10:1311.
7. Takagi, Y. [Home parenteral and enteral nutritional in Japan-present and future]. *Nihon Shokakibyo Gakkai Zasshi.* 2003;100:819–28.
8. Takagi, Y, A Okada *et al*. Report on the first annual survey of home parenteral nutrition in Japan. *Surg Today.* 1995;25:193–201.
9. Folwarski, M, S Kłęk *et al*. Trend Observations in Home Parenteral Nutrition. Prevalence, Hospitalizations and Costs: Results from a Nationwide Analysis of Health Care Provider Data. *Nutrients.* 2021;13:3465.
10. Wyszomirska, K, A Wyszomirski *et al*. Home Artificial Nutrition in Polish Children: An Analysis of 9-Year National Healthcare Provider Data. *Nutrients.* 2021;13:1007.
11. Smith, T, A Hirst *et al* (2011) *Artificial Nutition support in the UK 2000-2010*. Annual BANS report.
12. Smith, T and M Naghibi (2016) *British Artificial Nutrition Survey (BANS) Report 2016. Artificial Nutrition Support in the UK 2005-2015. Adult Home Parenteral Nutrition & Home Intravenous Fluids*.
13. Wiskin, AE, R Russell *et al*. Prevalence of home parenteral nutrition in children. *Clin Nutr ESPEN.* 2021;42:138–141.
14. Mundi, MS, A Pattinson *et al*. Prevalence of Home Parenteral and Enteral Nutrition in the United States. *Nutr Clin Pract.* 2017;32:799–805.
15. Winkler, MF, RA DiMaria-Ghalili *et al*. Characteristics of a Cohort of Home Parenteral Nutrition Patients at the Time of Enrollment in the Sustain Registry. *JPEN J Parentr Enteral Nutr.* 2016;40:1140–1149.
